# Radiological features of gait phenotypes in patients with idiopathic normal pressure hydrocephalus

**DOI:** 10.3389/fnagi.2025.1554642

**Published:** 2025-05-16

**Authors:** Silvia Nicolosi, Massimiliano Todisco, Matteo Paoletti, Eduardo Caverzasi, Francesco Tarantino, Elena Ballante, Francesca Valentino, Roberta Zangaglia, Silvia Figini, Giuseppe Cosentino, Claudio Pacchetti, Anna Pichiecchio

**Affiliations:** ^1^Department of Neuroradiology, Advanced Imaging Center and Artificial Intelligence, IRCCS Mondino Foundation, Pavia, Italy; ^2^Radiology Section, Department of Clinical-Surgical, Diagnostic, and Pediatric Sciences, University of Pavia, Pavia, Italy; ^3^Translational Neurophysiology Research Unit, IRCCS Mondino Foundation, Pavia, Italy; ^4^Movement Disorders Research Center, IRCCS Mondino Foundation, Pavia, Italy; ^5^Department of Brain and Behavioral Sciences, University of Pavia, Pavia, Italy; ^6^Department of Radiology, Di Summa-Perrino Hospital Center, Brindisi, Italy; ^7^BioData Science Center, IRCCS Mondino Foundation, Pavia, Italy; ^8^Department of Political and Social Sciences, University of Pavia, Pavia, Italy

**Keywords:** idiopathic normal pressure hydrocephalus, magnetic resonance imaging, gait disorder, parkinsonism, third ventricle

## Abstract

**Introduction:**

According to the higher-level gait disorder (HLGD) pattern, patients with idiopathic normal pressure hydrocephalus (iNPH) can be divided into two motor phenotypes; a disequilibrium (wide-based gait) subtype and a parkinsonian (locomotor) subtype. We aimed to understand the neuroimaging correlates of iNPH phenotyping into different gait patterns, by assessing specific radiological features and their correlations with clinical scores.

**Methods:**

We enrolled 86 probable iNPH patients (53 males; age range: 69–88 years), who underwent a comprehensive clinical assessment, including neuropsychological tests, and a conventional MRI scan. The cohort was subdivided into disequilibrium subtype (29 subjects) and parkinsonian subtype of HLGD (57 patients) based on gait evaluation. We compared the iNPH subtypes assessing differences in eight linear radiological indexes and their clinical correlates.

**Results:**

The Height of the third ventricle was the only radiological feature that differed between the two motor phenotypes (*p* < 0.05), being higher in the parkinsonian subtype and showing a trend of correlation with the motor score of the Movement Disorder Society-Unified Parkinson’s Disease Rating Scale and with the continence score of the iNPH Rating Scale. Among several clinical-radiological correlations, a reduced callosal angle correlated with the severity of motor and urinary symptoms (*p* < 0.05).

**Discussion:**

A greater height of the third ventricle possibly leading to a top-down compressive effect on the midbrain could be a neuroimaging marker of the parkinsonian phenotype of iNPH. The extensive correlations between linear radiological indices and clinical scales suggest a potential role for radiological features in clinical monitoring.

## 1 Introduction

Idiopathic normal pressure hydrocephalus (iNPH) is a complex cerebrospinal fluid dynamic disorder, clinically characterized by progressive motor, cognitive, and urinary disturbances due to ventricular enlargement, without any significant intracranial pressure abnormalities (Nakajima et al., 2021; Relkin et al., 2005). Despite representing one of the major neurological conditions that can benefit from surgical treatment (Nakajima et al., 2021; Todisco et al., 2020), iNPH is frequently underdiagnosed because subjects may have atypical clinical presentations beyond the classic triad of gait disorder, cognitive decline, and urinary incontinence (Todisco et al., 2019; Todisco et al., 2022).

The higher-level gait disorder (HLGD) represents the core clinical finding in iNPH (94–100% of cases), followed by cognitive impairment (78–98%) and urinary urgency (60–92%) (Relkin et al., 2005). However, gait disturbance of iNPH shows high variability of phenomenology. Indeed, if on the one hand the typical gait pattern is characterized by wide base with externally rotated feet and impaired balance with falls, on the other hand about two-thirds of patients can present parkinsonian features, i.e., slowness, shuffling steps, freezing of gait, and *en bloc* turning (Todisco et al., 2021; Pozzi et al., 2021). This evidence led to the dichotomous classification into a disequilibrium (wide-based gait) subtype, defined as phenotype 1, and a parkinsonian subtype, known as phenotype 2, respectively (Todisco et al., 2021; Pozzi et al., 2021; Nutt, 2013).

Brain magnetic resonance imaging (MRI) plays an essential role in the diagnosis of iNPH, since it provides routinely used radiological indexes—as Evans’ Index (EI) and callosal angle (CA) at the level of the posterior commissure—and also assists in patient stratification into the diagnostic categories of “possible” or “probable” iNPH according to the Japanese Guidelines, based on the evaluation of Disproportionately Enlarged Subarachnoid Space Hydrocephalus (DESH) (Nakajima et al., 2021; Relkin et al., 2005). To date, radiological underpinnings of the clinical symptoms in iNPH are still matter of debate. Neuroimaging correlates of the distinct phenotypes of HLGD have not yet been investigated. In this study, we aimed to apply several radiological indexes used for diagnostic and research purposes to assess differences between the two motor phenotypes of iNPH. Moreover, we explored potential correlations of MRI features with clinical severity scores.

## 2 Materials and methods

### 2.1 Study subjects

As part of a prospective longitudinal evaluation, between January 2016 and December 2018 we consecutively recruited 86 patients diagnosed as “probable” iNPH according to the International Guidelines and admitted to the Parkinson’s Disease and Movement Disorders Unit of the Mondino Foundation in Pavia (Italy) (Nakajima et al., 2021). All subjects were clinically assessed by a movement disorder specialist, who qualitatively established the motor phenotype based on the HLGD pattern as previously detailed (Todisco et al., 2021; Pozzi et al., 2021). No patient was under diuretic treatment. A second blinded clinician reevaluated the gait subtype of iNPH patients, showing a full agreement. The clinical and neuropsychological evaluation included the iNPH Rating Scale (iNPHRS) (Hellström et al., 2012), the motor section of the Movement Disorder Society-Unified Parkinson’s Disease Rating Scale (MDS-UPDRS III) (Goetz et al., 2008) and the education-adjusted scores of the Mini Mental State Examination and of the Montreal Cognitive Assessment (MoCA).

Within six months from the neurological assessment, all patients underwent a 1.5 or 3 Tesla brain MRI scan, including routine sequences for the diagnostic work-up, such as axial fluid-attenuated inversion recovery (FLAIR), coronal and sagittal T2-weighted turbo spin-echo (TSE), and T1-weighted gradient echo (GRE) three-dimensional sequences with multiplanar reconstructions, at the Neuroradiology Unit of the same hospital.

The Ethics Committee of the Mondino Foundation approved the study. All patients gave written informed consent to all study procedures and to personal data processing for research purposes, according to the Declaration of Helsinki. The study protocol was entirely performed before eventual shunt surgery treatment.

### 2.2 Neuroimaging measurements

**FIGURE 1:**
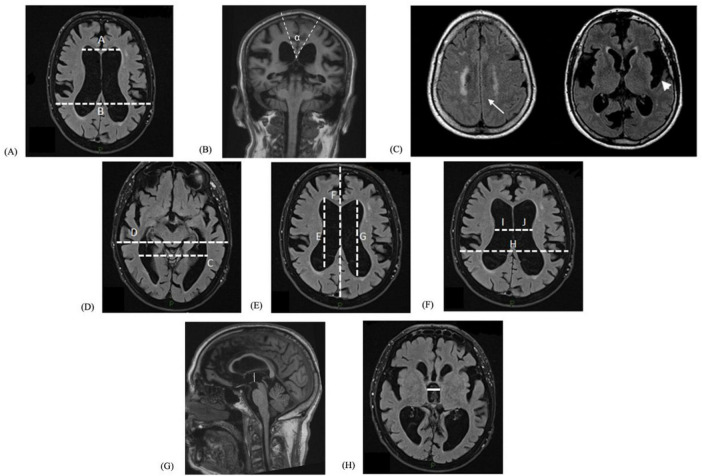
Linear radiological indexes. **(A)** Evans’ Index: maximal width of the frontal horns (A) and the largest internal diameter of the cranium at the same slice (B). **(B)** Callosal angle: the angle is calculated at the level of posterior commissure (α). **(C)** Disproportionately Enlarged Subarachnoid Space Hydrocephalus: narrowing of the subarachnoid spaces near the vertex (arrow) and widening of the Sylvian fissures (arrowhead). The hyperintense periventricular white matter abnormalities in both axial FLAIR sequences are referred to slight chronic microvascular damage. **(D)** Magnetic Resonance Hydrocephalic Index: the largest left-to-right width of the collateral trigones of the lateral ventricles (C) and maximum inner skull diameter (D). **(E)** Anteroposterior diameter of the Lateral Ventricle Index, evaluated for each side: anteroposterior diameter of each lateral ventricle (E and G, right and left, respectively) and the maximal width of the anteroposterior inner diameter of the skull (along the cerebral falx) (F). **(F)** Modified Cella Media Index, assessed for both sides: the largest internal diameter of the cranium (H) and the maximum cella media width of each lateral ventricle (I and J, right and left, respectively). **(G)** Height of the third ventricle. **(H)** Width of the third ventricle.

Blinded to patients’ clinical examination, an expert neuroradiologist (with ten years of experience in the field) reviewed all brain MRI scans, assessing eight quantitative linear radiological indexes, so as to remove the inter-rater variability. The radiological indexes included: EI (Figure 1A); CA (Figure 1B); DESH (Figure 1C); Magnetic Resonance Hydrocephalic Index (MRHI) (Figure 1D); Anteroposterior diameter of the Lateral Ventricle Index (ALVI) (Figure 1E); modified Cella Media Index (mCMI) (Figure 1F); and Height and Width of the third ventricle (IIIvH and IIIvLL, respectively) (Figures 1G, H). EI is the ratio between the maximal width of the frontal horns and the largest internal diameter of the cranium at the same slice. An EI greater than 0.30 is widely used to determine an enlargement of the supratentorial ventricular system (Nakajima et al., 2021; Relkin et al., 2005). CA is measured on the coronal plane through the posterior commissure perpendicular to the anterior-commissural line (AC-PC line). A CA value lower than 90–100° is considered highly accurate to discriminate iNPH patients from healthy controls or patients with Alzheimer’s disease, as lower values were found in iNPH (Ishii et al., 2008). DESH is considered present in case of narrowing of the high-convexity and medial subarachnoid spaces with enlarged Sylvian fissures on T1-weighted coronal images (Relkin et al., 2005). MRHI is obtained by dividing the collateral trigones width by the internal diameter of the cranial theca, as previously proposed by Quattrone et al. (2020). ALVI is calculated as the ratio between the width from the most anterior to the most posterior points of the lateral ventricles and the sagittal diameter of the internal cranial theca at the same level (He et al., 2020). mCMI is defined as the ratio between the maximal width of the cella media and the maximal inner diameter of the skull at the same level on an axial plane (Kojoukhova et al., 2015). Both ALVI and mCMI were assessed for each side separately (right and left, i.e., ALVI R/L and mCMI R/L). IIIvH and IIIvLL are calculated as the diameter from the roof to the floor of the third ventricle perpendicular to the AC-PC line and as the greatest latero-lateral diameter of the third ventricle perpendicular to the median line, respectively (Reinard et al., 2015). By means of ruler and angle measurement available on the routine neuroimaging viewing system, the radiological measures were manually taken in the axial plane, except for the CA, calculated on a paracoronal plane perpendicular to the AC-PC line, and the IIIvH, evaluated on sagittal sequences (Reinard et al., 2015).

### 2.3 Statistical analysis

Statistical analysis was performed using R software version 4.4.2. The Wilcoxon test was used to explore any difference between two motor phenotypes of HLGD, except for the binary categorical variables gender and DESH for which Pearson’s chi-squared test with Yates’ continuity correction was applied. Considering the small sample size and the high number of correlations, a multiple comparison correction was not applied. For each correlation, the Cohen’s d effect size was calculated to measure the strength of the relationship between variables and reported in conjunction with *p*-values. The magnitude of the effect size should be assessed using the thresholds provided in Cohen, 1992, i.e., |d| < 0.2 “negligible,” |d| < 0.5 “small,” |d| < 0.8 “medium,” otherwise “large.” Comparisons between radiological indices and clinical scores were calculated using Spearman’s rank correlation test, already a measure of the effect, whereas for DESH the Wilcoxon test with continuity correction and relative Cohen’s D effect size were used. Statistical significance was defined for *p*-values below 0.05.

## 3 Results

### 3.1 Demographics, clinical scores and MRI indexes

Demographic, clinical and radiological data for the entire cohort and for each motor phenotype of HLGD are reported in Table 1. Within the whole cohort of 86 iNPH patients, we identified 29 subjects (34%) with phenotype 1 (disequilibrium subtype) and 57 subjects (66%) with phenotype 2 (parkinsonian subtype). The two motor phenotypes did not differ significantly in demographic variables. Conversely, patients with phenotype 2 showed more severe parkinsonism (higher MDS-UPDRS III score), more impaired gait and balance (lower iNPHRS scores at the related items), worse cognitive scores (lower MMSE and MoCA scores), and poorer global functioning (lower iNPHRS total score).

**TABLE 1:** Demographics, clinical data and MRI features of iNPH patients.

	Entire cohort (*n* = 86)	Phenotype 1 (*n* = 29)	Phenotype 2 (*n* = 57)	Effect size	*P*-values
Age (years)	75 ± 3	76 ± 5	76 ± 6	0.091	0.392
Age at motor symptoms onset (years)	73 ± 6	73 ± 6	73 ± 7	0.028	**0.833**
Gender (M/F)	53/33	18/11	35/22	0	1
MDS-UPDRS III	19 ± 13	8 ± 4	25 ± 12	**1.682**	**< 0.001**
iNPHRS gait	42 ± 23	54 ± 22	36 ± 22	**0.798**	**< 0.001**
iNPHRS balance	58 ± 17	65 ± 14	55 ± 17	**0.644**	**0.002**
iNPHRS continence	66 ± 23	72 ± 25	62 ± 21	0.458	0.051
iNPHRS total	48 ± 17	57 ± 16	44 ± 15	**0.868**	**< 0.001**
MMSE	23 ± 6	25 ± 4	22 ± 7	**0.549**	**0.035**
MoCA	17 ± 6	20 ± 6	16 ± 6	**0.678**	**0.015**
EI	0.35 ± 0.04	0.35 ± 0.03	0.36 ± 0.04	0.160	0.967
CA (°)	84.39 ± 18.04	89.20 ± 23.03	81.70 ± 18.33	0.374	0.225
DESH (%)	80	81	79	0	1
MRHI	0.57 ± 0.03	0.58 ± 0.04	0.58 ± 0.03	0.109	0.559
ALVI R	0.52 ± 0.04	0.53 ± 0.04	0.54 ± 0.05	0.314	0.466
ALVI L	0.53 ± 0.04	0.54 ± 0.05	0.55 ± 0.05	0.173	0.690
mCMI R	0.16 ± 0.02	0.17 ± 0.02	0.17 ± 0.02	0.183	0.315
mCMI L	0.16 ± 0.03	0.18 ± 0.02	0.17 ± 0.03	0.397	0.065
IIIvH	13.99 ± 1.65	13.40 ± 1.55	14.3 ± 1.62	**0.593**	**0.017**
IIIvLL	13.36 ± 2.81	13.60 ± 2.70	13.20 ± 2.88	0.132	0.304

Findings from the entire cohort and from each motor phenotype, expressed as mean ± standard deviation or as numbers or percentages. Statistical *p*-values and Cohen’s d effect sizes of comparisons between the two motor phenotypes are reported. Statistically significance values are in bold. ALVI, Anteroposterior diameter of the Lateral Ventricle Index; CA, callosal angle; DESH, Disproportionately Enlarged Subarachnoid Space Hydrocephalus; EI, Evans’ Index; iNPH, idiopathic normal pressure hydrocephalus; iNPHRS, iNPH Rating Scale; L, left; mCMI, modified Cella Media Index; MDS-UPDRS III, motor score of the Movement Disorder Society-Unified Parkinson’s Disease Rating Scale; MMSE, Mini Mental State Examination; MoCA, Montreal Cognitive Assessment; MRHI, Magnetic Resonance Hydrocephalic Index; R, right; IIIvH, height of the third ventricle; IIIvLL, width of the third ventricle.

**FIGURE 2:**
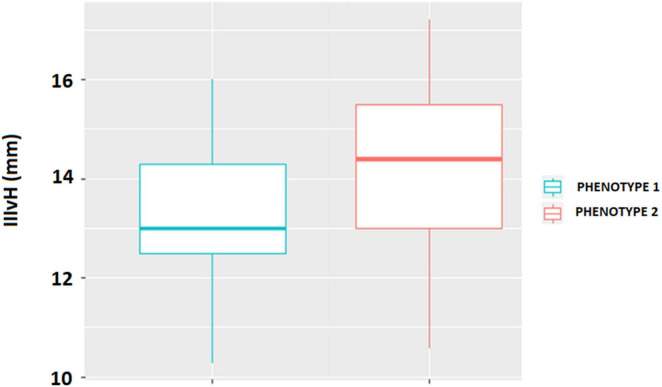
Comparison of the Height of the third ventricle between phenotype 1 (disequilibrium subtype) and phenotype 2 (parkinsonian subtype) of higher-level gait disorder. The difference was explored with the Wilcoxon test (*p* = 0.017).

Among the radiological measures, IIIvH was the only index that differed significantly between the two motor phenotypes, given higher values in patients with phenotype 2 (Figure 2).

### 3.2 Clinical correlations of MRI indexes

Correlation analyses between neuroimaging indices and clinical scores in the entire patients’ cohort are detailed in Table 2, several of these were significant (*p*-values below 0.05). EI showed a negative correlation with the iNPHRS total score (including its gait and continence domains). We found a positive correlation of CA with the iNPHRS total score and its gait, balance and continence domains. By contrast, CA negatively correlated with the MDS-UPDRS III score. ALVI R/L showed a negative correlation with the iNPHRS total score and its gait and balance domains, while only ALVI L negatively correlated with the continence domain and only ALVI R positively correlated with the MDS-UPDRS III score. MRHI and both ALVI R/L were the only indices that negatively correlated with the MoCA score. We did not find any significant correlation of clinical scores with DESH, mCMI R/L, IIIvH, and IIIvLL. Nonetheless, IIIvH showed trends of correlation with the severity of parkinsonism and urinary impairment (MDS-UPDRS III and iNPHRS continence scores, respectively) (Supplementary Figure 1).

**TABLE 2:** Correlations between radiological indices and clinical scores in the entire patients’ cohort.

	MDS -UPDRS III	iNPHRS gait	iNPHRS balance	iNPHRS continence	iNPHRS total	MMSE	MoCA
EI	0.134	**−0.250** *	−0.198	**−0.264** *	**−0.270** *	−0.129	−0.229
CA	**−0.0279** *	**0.359** ***	**0.357** ***	**0.288** **	**0.320** **	0.102	0.106
DESH	482 (0.256)	652 (0.262)	615.5 (0.125)	657.5 (0.208)	640 (0.212)	376 (0.255)	349 (0.203)
MRHI	0.185	−0.188	−0.160	0.031	−0.0124	−0.212	**−0.372** **
ALVI R	**0.009**	**−0.293** **	**−0.266** *	−0.173	**0.008**	−0.232	**−0.334** **
ALVI L	0.087	**−0.259** *	**−0.304** **	**−0.251** *	**0.008**	−0.191	**−0.297** *
mCMI R	−0.039	0.058	−0.014	−0.0923	0.004	0.098	−0.048
mCMI L	−0.015	0.034	−0.135	0.065	−0.018	0.113	−0.106
IIIvH	0.203	−0.010	−0.113	−0.198	−0.115	0.199	0.177
IIIvLL	−0.069	0.060	−0.068	−0.133	−0.047	0.316	0.907

Statistical correlations between linear radiological features and clinical scales are assessed by Spearman correlation test, except for DESH by the Wilcoxon rank sum test with continuity correction with Cohen’s d effect size in brackets. Statistically significance values are in bold.

*p-value < 0.05;

**p-value < 0.01;

***p-value < 0.001. ALVI, Anteroposterior diameter of the Lateral Ventricle Index; CA, callosal angle; DESH, Disproportionately Enlarged Subarachnoid Space Hydrocephalus; EI, Evans’ Index; iNPH, idiopathic normal pressure hydrocephalus; iNPHRS, iNPH Rating Scale; L, left; mCMI, modified Cella Media Index; MDS-UPDRS III, motor score of the Movement Disorder Society-Unified Parkinson’s Disease Rating Scale; MMSE, Mini Mental State Examination; MoCA, Montreal Cognitive Assessment; MRHI, Magnetic Resonance Hydrocephalic Index; R, right; IIIvH, height of the third ventricle; IIIvLL, width of the third ventricle.

Clinical-radiological correlations for each motor phenotype are reported in Supplementary Tables 1, 2 (phenotype 1 and phenotype 2, respectively). A negative correlation of EI with the iNPHRS total score (and its gait and continence domains) was confirmed only in the phenotype 1, where EI also showed a positive correlation with the MDS-UPDRS III score. A positive correlation of CA with the iNPHRS total score (and its balance and continence domains) was found in the phenotype 2. The negative correlation of MRHI with the MoCA score was found in the phenotype 2, where MRHI also negatively correlated with the iNPHRS balance score. Conversely, MRHI positively correlated with the MDS-UPDRS III score in the phenotype 1. The negative correlation of ALVI R/L with the iNPHRS total score (as well as its gait item) was shown in the phenotype 1, while ALVI R/L negatively correlated with the balance domain in the phenotype 2. Of note, ALVI R/L positively correlated with the MDS-UPDRS III score in both phenotypes. IIIvLL negatively correlated with the iNPHRS total score and positively correlated with the MDS-UPDRS III score in the phenotype 1. As in the entire cohort, we did not find any statistically significant correlation of clinical scores with DESH, mCMI R/L, and IIIvH for both phenotype 1 and 2.

## 4 Discussion

In this study, we explored differences in eight linear MRI indexes and their clinical correlations between the two motor phenotypes of iNPH. IIIvH was the only neuroimaging feature that differed between the two motor phenotypes, being higher in iNPH patients with parkinsonian subtype of HLGD who showed also a greater global clinical impairment in terms of severity of motor, urinary and cognitive dysfunction, compared to subjects with disequilibrium subtype. It is also worth noting that, although not statistically significant, the clinical relevance of IIIvH may also be supported by trends in correlation with overall parkinsonian and urinary symptom burden.

The pathophysiology of parkinsonian symptoms in hydrocephalic state is still unclear, probably multifactorial. It has been hypothesized that an increased IIIvH may cause a direct top-down compression on the rostral midbrain, resulting in a reduced mesencephalic volume. In iNPH patients, the midbrain diameter, in fact, was found to be decreased, showing an inverse correlation with the width of the third ventricle and the severity of gait disturbance (Lee et al., 2005). Alternatively, the midbrain compression deriving from a dilated third ventricle may cause a chronic deafferentation (Lee et al., 2005), leading a dysfunction of neuronal groups of specific mesencephalic areas that are crucial for the control of gait and balance, such as the pedunculopontine nucleus, generating a phenotype similar to that observed in Parkinson’s disease (Sébille et al., 2019; Lee et al., 2000). The substantia nigra is another midbrain nucleus whose impairment may be involved in the pathophysiology of parkinsonism frequently observed in iNPH (Youn et al., 2022). Of note, density of striatal dopamine reuptake transporter was described to be abnomal in iNPH, and particularly lower in patients with parkinsonian subtype as compared to subjects with disequilibrium subtype of HLGD (Todisco et al., 2021; Pozzi et al., 2021). Furthermore, the impairment of the nigrostriatal dopaminergic function correlated with the severity of parkinsonism (Todisco et al., 2021; Pozzi et al., 2021). An alteration of the nigrostriatal system due to an expanding IIIv has been also suggested in some cases of different non-communicating forms of hydrocephalus and secondary parkinsonism as well, also due to acqueductal stenosis, as shown in the literature (Lee et al., 2005; Curran and Lang, 1994; Zeidler et al., 1998).

A midbrain dysfunction could contribute to impair urinary storage function in iNPH. The periaqueductal gray matter is a key mesencephalic area for micturition control, being connected both to upstream cortical and diencephalic centers (e.g., prefrontal, cingulate and insular cortex, thalamus and medial preoptic area of hypothalamus) and to downstream pontine and sacral spinal segments (e.g., Barrington’s nucleus and Onuf’s nucleus) that regulate bladder function (Zare et al., 2019). It has been suggested to be affected in iNPH, given the suggestion that a dysfunction of this midbrain area may contribute to determine small bladder capacity and detrusor overactivity observed in these patients (Sakakibara et al., 2008).

We found that a higher EI, a reduced CA, and a greater ALVI R/L were relevant radiological features associated with the severity of motor and urinary symptoms in our patients’ cohort. Notably, CA was the single index showing the most significant values at correlation analyses with clinical scores, in keeping with other authors (Kockum et al., 2018), also considering its recently proved reliability in follow-up after shunt surgery, where an increase in CA was observed alongside neurologic improvement (Casimiro Reis et al., 2024). Considering that these linear radiological measures reflect morphology and volume of the lateral ventricles, it is reasonable to assume that the ventricular dilatation at this level can account for urinary dysfunction and multiple aspects of motor impairment, involving gait, balance, and appendicular movements possibly due to white matter disorders as detected by DTI technique (Marumoto et al., 2012). On a histological level, these patients present focal destruction of the ependyma with distortion and collapse of capillaries and chronic oedema or ischemic demyelination (Del Bigio, 1993), however, whether the ventricular dilatation is the *primum movens* or rather an epiphenomenon of an alternative pathophysiological mechanism remains to be demonstrated.

In parallel, we detected an increase of MRHI and ALVI R/L in association with the severity of cognitive decline, assessed by the MoCA score. These findings can emphasize the role of periventricular regions known to be involved in several associative cognitive functions, in particular corpus callosum and cingulum, indirectly measured by ALVI R/L, and arcuate fasciculus, indirectly measured by MRHI (Güngör et al., 2017; Lin et al., 2014). It could be suggested that transependymal diffusion, oedema, and stretch or compression of these periventricular regions may be engaged in cognitive deficits of iNPH patients, as confirmed by a tractography study (Keong et al., 2017).

On the one hand, mCMI R/L and DESH did not disclose any correlation with the clinical scores. It could be argued that mCMI R/L is not as sensitive as other radiological indices in quantifying the extent of ventricular dilatation, while the qualitative observation of DESH in the majority of iNPH patients does not allow adequate investigation of the links to the multifaceted phenomenology of these patients. With this regard, the quantitative assessment of DESH could be desirable to improve the chance to detect clinical-radiological correlations. On the other hand, and differently from MoCA, MMSE score did not correlate with any radiological index. The report of high sensitivity, lack of ceiling effect, and good detection of cognitive heterogeneity could make MoCA a better measure of cognitive function as compared to MMSE (Jia et al., 2021), and thus potentially preferable to improve the cognitive characterization of iNPH patients and to effectively assess correlations with neuroimaging features.

The intra-group analyses showed that motor phenotypes were featured by different correlations of radiological indexes with clinical scores. In fact, EI, MRHI, and IIIvLL correlated with several motor and cognitive scores in patients with disequilibrium subtype, whereas MRHI was valuable measure in patients with parkinsonian subtype being correlated with balance impairment and cognitive severity. Instead, only ALVI R/L correlated with several motor scores in both gait subtypes. Once again, the peculiar radiological characterization of each motor phenotype of HLGD could be explained by distinct pathophysiological basis underlying the heterogenous phenomenological expression of iNPH.

The study has several limitations. Firstly, the small cohort of patients and the inhomogeneity of the sample due to the definite prevalence of the parkinsonian motor phenotype must be considered. Furthermore, due to the high number of correlations and the exploratory nature of the study, a multiple comparison was not feasible, although this model would have taken into account additional inter- and intra-individual clinical and anatomical variables that might influence these correlations.

Those linear radiological indices, even if more practical and applicable to CT images, may be less accurate than volumetric analysis, suggesting the need for further validation through brain 3D measurements as well. Although, our findings suggest the possible role of these neuroimaging markers in clinical practice for supporting diagnosis and for monitoring the clinical evolution of the disease, also over time. The radiological characterization of motor phenotypes of iNPH may benefit from further investigation using tractography or other advanced MRI imaging techniques i.e., functional MRI or multicompartmental diffusion model imaging to understand the underlying pathophysiology (Keong et al., 2017; Siasios et al., 2016).

## Data Availability

The datasets presented in this study can be found in online repositories. The names of the repository/repositories and accession number(s) can be found in this article/Supplementary material.
