# Supplementary material for: Radiological features of gait phenotypes in patients with idiopathic normal pressure hydrocephalus
Source: Front Aging Neurosci. 2025 May 16;17:1554642. doi: 10.3389/fnagi.2025.1554642 (PMC12122746; doi:10.3389/fnagi.2025.1554642)
Supplement: Supplementary file 1 [file Data_Sheet_1.pdf]

Supplementary Material

1 Supplementary Tables

Supplementary Table 1. Correlations between radiological features and clinical scores in patients with phenotype 1 of higher-level gait disorder.

Significant coefficients are emphasized in bold, assessed by Spearman test except for DESH by the Wilcoxon rank sum test with continuity correction.

*p-value < 0.05 **p-value < 0.01 *p-value < 0.001.

Abbreviations: ALVI, Anteroposterior diameter of the Lateral Ventricle Index; CA, Callosal Angle; DESH, Disproportionately Enlarged Subarachnoid Space Hydrocephalus; EI, Evans' Index; iNPH, idiopathic normal pressure hydrocephalus; iNPHRS, iNPH Rating Scale; L, left; mCMI, modified Cella Media Index; MDS-UPDRS III, Movement Disorder Society-Unified Parkinson's Disease Rating Scale; MMSE, Mini Mental State Examination; MoCA, Montreal Cognitive Assessment; MRHI, Magnetic Resonance Hydrocephalic Index; R, right; IIIvH, Height of the Third Ventricle; IIIvLL, Width of the Third Ventricle.

	**MDS-UPDRS III**	**iNPHRS gait**	**iNPHRS balance**	**iNPHRS continence**	**iNPHRS total**	**MMSE**	**MoCA**
**EI**	**0.429***	**-0.455***	-0.301	**-0.494****	**-0.573*****	-0.362	-0.373
**CA**	-0.370*	0.359	0.311	0.249	0.251	-0.021	-0.007
**DESH**	65	89	67.5	76	86	55	50
**MRHI**	**0.408***	-0.296	-0.052	-0.007	-0.249	-0.119	-0.248
**ALVI R**	**0.547****	**-0.481****	-0.287	-0.359	**-0.471****	-0.247	-0.338

**ALVI L**	**0.460***	**-0.379***	-0.247	-0.349	**-0.406***	-0.214	-0.247
**mCMI R**	0.130	-0.123	0.072	-0.225	-0.097	-0.053	-0.039
**mCMI L**	**0.515****	-0.223	-0.249	-0.234	-0.303	0.098	0.021
**IIIvH**	0.030	0.101	-0.113	-0.343	-0.102*	0.031	-0.041
**IIIvLL**	**0.397***	-0.290	-0.339	-0.288	**-0.376***	0.046	-0.046

Supplementary Table 2. Correlations between radiological features and clinical scores in patients with phenotype 2 of higher-level gait disorder.

Significant p-values are emphasized in bold. *p-value < 0.05 **p-value < 0.01 ***p-value < 0.001

Abbreviations: ALVI, Anteroposterior diameter of the Lateral Ventricle Index; CA, Callosal Angle; DESH, Disproportionately Enlarged Subarachnoid Space Hydrocephalus; EI, Evans' Index; iNPH, idiopathic normal pressure hydrocephalus; iNPHRS, iNPH Rating Scale; L, left; mCMI, modified Cella Media Index; MDS-UPDRS III, Movement Disorder Society-Unified Parkinson's Disease Rating Scale; MMSE, Mini Mental State Examination; MoCA, Montreal Cognitive Assessment; MRHI, Magnetic Resonance Hydrocephalic Index; R, right; IIIvH, Height of the Third Ventricle; IIIvLL, Width of the Third Ventricle.

	**MDS-UPDRS III**	**iNPHRS gait**	**iNPHRS balance**	**iNPHRS continence**	**iNPHRS total**	**MMSE**	**MoCA**
**EI**	0.170	0.897	-0.199	-0.132	-0.081	0.219	-0.022

**CA**	**-0.223**	0.244	**0.391****	**0.317***	**0.279***	0.086	0.080
**DESH**	181	270.5	282.5	285.5	262	143	130
**MRHI**	0.226	-0.097	**-0.341****	0.034	-0.117	-0.158	**-0.365***
**ALVI R**	**0.318***	-0.157	**-0.333***	-0.057	-0.218	-0.083	-0.222
**ALVI L**	**0.307***	-0.153	**-0.407****	-0.179	-0.257	0.008	-0.172
**mCMI R**	0.002	0.100	-0.086	-0.051	0.002	0.128	-0.103
**mCMI L**	0.084	0.030	-0.190	0.141	-0.022	0.058	-0.264
**IIIvH**	0.048	0.095	-0.005	-0.037	0.034	-0.121	-0.074
**IIIvLL**	-0.117	0.189	0.001	-0.074	0.073	0.146	-0.015

2 Supplementary Figure

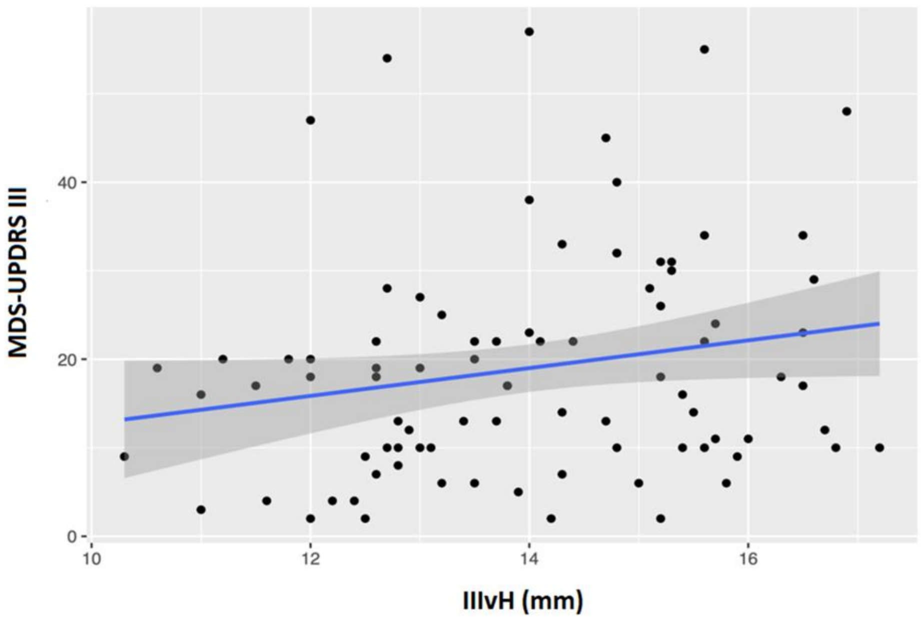
MDS-UPDRS III
0
20
40
10
12
14
16
IIIvH (mm)

(A)

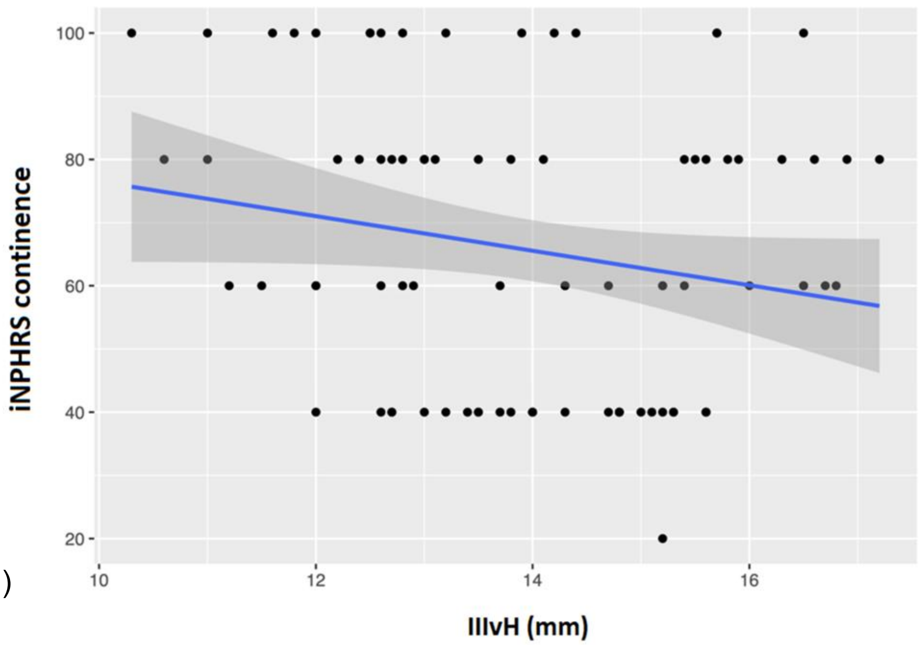
iNPHRS continence
20
40
60
80
100
10
12
14
16
IIIvH (mm)

(B)

Supplementary Fig. 1. Correlations of IIIvH with MDS-UPDRS III (A) and iNPHRS continence item (B) in the entire patients' cohort, not significant at 5% but at 10%. Abbreviations: iNPHRS, iNPH Rating Scale; MDS-UPDRS III, motor score of the Movement Disorder Society-Unified Parkinson's Disease Rating Scale; IIIvH, Height of the Third Ventricle.
